# Building a stakeholder-led common vision increases the expected cost-effectiveness of biodiversity conservation

**DOI:** 10.1371/journal.pone.0218093

**Published:** 2019-06-13

**Authors:** Rocío Ponce Reyes, Jennifer Firn, Sam Nicol, Iadine Chadès, Danial S. Stratford, Tara G. Martin, Stuart Whitten, Josie Carwardine

**Affiliations:** 1 CSIRO Land and Water, EcoSciences Precinct, Brisbane, Queensland, Australia; 2 Queensland University of Technology, Gardens Point Campus, Brisbane, Queensland, Australia; 3 CSIRO Land and Water, Canberra, Australian Capital Territory, Australia; 4 Department of Forest & Conservation Sciences, University of British Columbia, Vancouver, Canada; University of Waikato, NEW ZEALAND

## Abstract

Uniting diverse stakeholders through communication, education or building a collaborative ‘common vision’ for biodiversity management is a recommended approach for enabling effective conservation in regions with multiple uses. However, socially focused strategies such as building a collaborative vision can require sharing scarce resources (time and financial resources) with the on-ground management actions needed to achieve conservation outcomes. Here we adapt current prioritisation tools to predict the likely return on the financial investment of building a stakeholder-led vision along with a portfolio of on-ground management strategies. Our approach brings together and analyses expert knowledge to estimate the cost-effectiveness of a common vision strategy and on-ground management strategies, before any investments in these strategies are made. We test our approach in an intensively-used Australian biodiversity hotspot with 179 threatened or at-risk species. Experts predicted that an effective stakeholder vision for the region would have a relatively low cost and would significantly increase the feasibility of on-ground management strategies. As a result, our analysis indicates that a common vision is likely to be a cost-effective investment, increasing the expected persistence of threatened species in the region by 9 to 52%, depending upon the strategies implemented. Our approach can provide the maximum budget that is worth investing in building a common vision or another socially focused strategy for building support for on-ground conservation actions. The approach can assist with decisions about whether and how to allocate scarce resources amongst social and ecological actions for biodiversity conservation in other regions worldwide.

## Introduction

More than 75% of the planet’s terrestrial ecosystems have been altered by human activities since the industrial revolution [1]. Today, remnant high conservation value ecosystems are embedded in mosaics of different land-uses and the number of species threatened with extinction is accelerating [2, 3]. Finding solutions to avoid further losses of ecosystems and native species is more difficult in regions with multiple land-use activities because challenges such as insufficient ongoing resources for long-term management implementation, local stakeholders not supporting top-down management decisions, inadequate information for making management decisions and conflicts between land-use for development, production and nature conservation may be more acute [4–6].

A strategy for managing complex multi-use regions is building a collaborative stakeholder-led ‘common vision’ for biodiversity conservation management, to align disparate goals and management in the region [7–9]. A common or shared vision, developed through stakeholder collaboration, describes an attractive or acceptable future to the collective of people involved in its development [10]. Broad stakeholder involvement in the management of natural resources strengthens democratic cultures and processes [11], provides additional knowledge and values for decision-making [12], increases legitimacy [13] and trust [14], and reduces conflicts [15]. Environmentally-oriented common visions have been built in different parts of the world in attempts to reconcile diverse values, set shared goals, balance competing interests and identify pathways for implementing on-ground strategies for managing regional assets, including biodiversity [16].

Stakeholder collaborations have resulted in perceived and measurable benefits to ecological outcomes, including biodiversity, by improving the decision-making and management [17]. Statements around the importance of stakeholder collaboration, communication and building shared goals, are commonplace in planning documents. However, there are non-negligible costs associated with embarking on collaborative planning processes (e.g. staff time and travel)[18], as well as risks of failure. These risks include an inability to reach a shared vision due to extremely contrasting views [9], unclear accountability or inefficient resource use [19]. While a small number of previous studies have carried out retrospective evaluations of the benefits of stakeholder involvement and collaborations, the costs, and therefore, the return on investment of the resources spent on developing and implementing a shared vision have not been quantified [20].

The benefits of overarching or enabling strategies, such as forming collaborations, governance bodies or information sharing networks, or investing in capacity building, education and outreach, are rarely quantified separately to on-ground actions [17, 21]. This limits our understanding on whether the benefits generated by the collaboration are a worthwhile investment, and how much we should invest in them, particularly when finite resources must be shared amongst all the necessary actions for achieving threatened species and ecosystem management and recovery.

**Fig 1:**
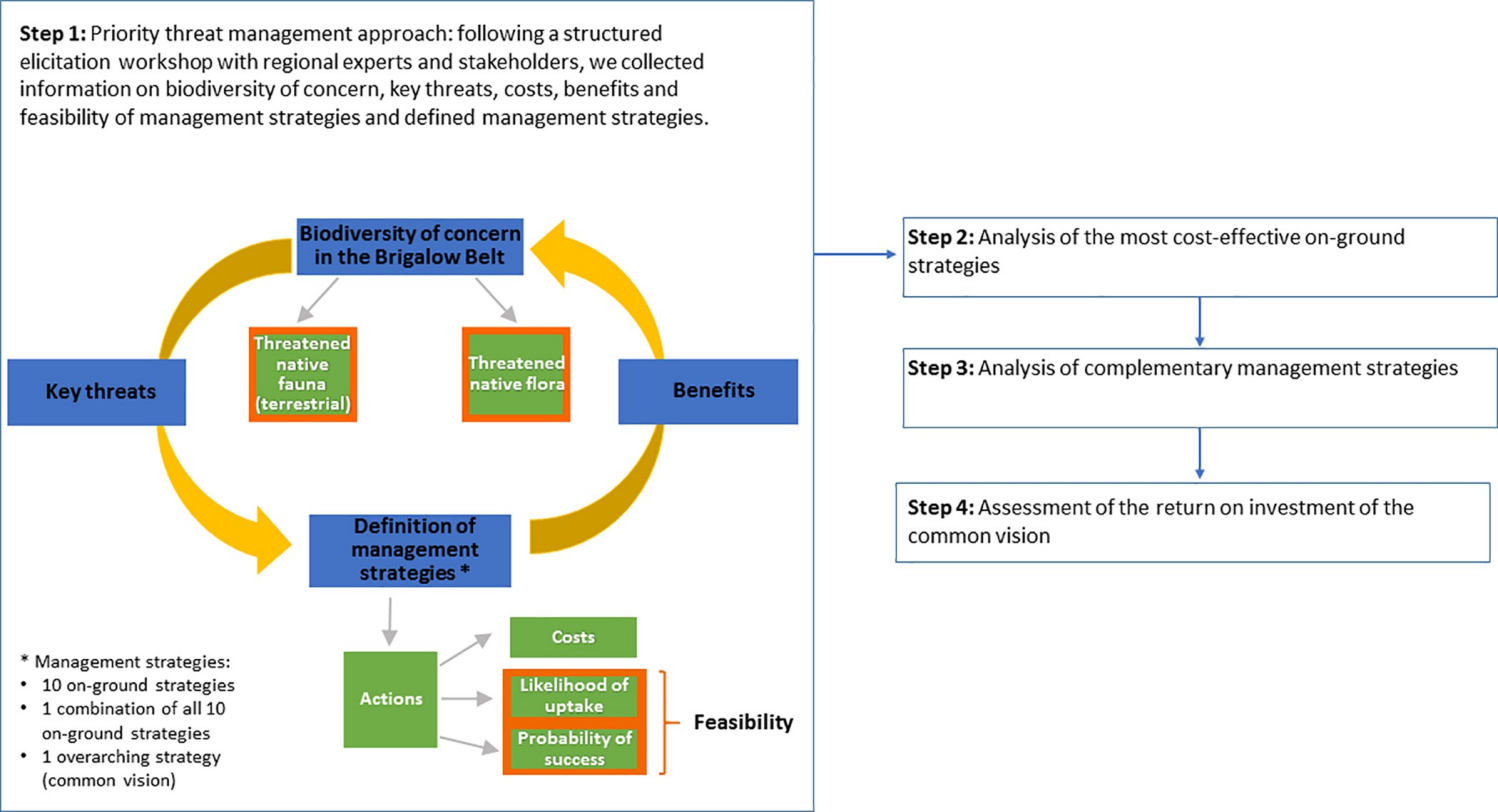
Diagram of the methods adopted in this study.

Here, we propose a decision science approach to estimate, at the prioritisation stage, the cost-effectiveness of investing in a common vision that consolidates stakeholder values and guides management priorities and implementation (Fig 1). We applied our approach to a case study with a full portfolio of on-ground conservation management strategies to protect threatened species, using a Priority Threat Management analysis of one of Australia’s most transformed, yet biodiversity-rich regions; the Queensland Brigalow Belt [22]. Priority Threat Management identifies the most cost-effective management actions across land tenures to maximise the benefits to biodiversity, using structured elicitation to collate information on costs, benefits and feasibility of each action [23, 24]. Previously this approach has been applied in relatively ecologically intact, sparsely populated regions of Australia [23, 25–28] and Canada [29]. None of these studies, however, considered the cost-effectiveness of investing in a common vision.

The aims involved with this study were to: (i) collect expert information from the region, including key threats to the threatened or at-risk species in the Brigalow Belt bioregion, management strategies that would improve the functional persistence of threatened species, cost and feasibility of implementing the management strategies and the potential benefits to the probability of persistence of threatened species if the strategies were implemented;(ii) analyse the expected cost-effectiveness of each of the on-ground management strategies in increasing total threatened species persistence per dollar spent, over the next 50 years in the Brigalow Belt region; (iii) identify complementary sets of on-ground strategies that maximise the expected persistence of as many threatened species as possible over the 50-year term;(iv) assess the expected improvements in cost-effectiveness generated by implementing a common vision (as defined by the participants) alongside the portfolio of on-ground management activities; and finally, (v) determine a threshold or breakeven value for how much funding could be spent on building the common vision within a cost-effective investment portfolio for the Brigalow Belt Bioregion.

## Material and methods

### Case study region

**Fig 2:**
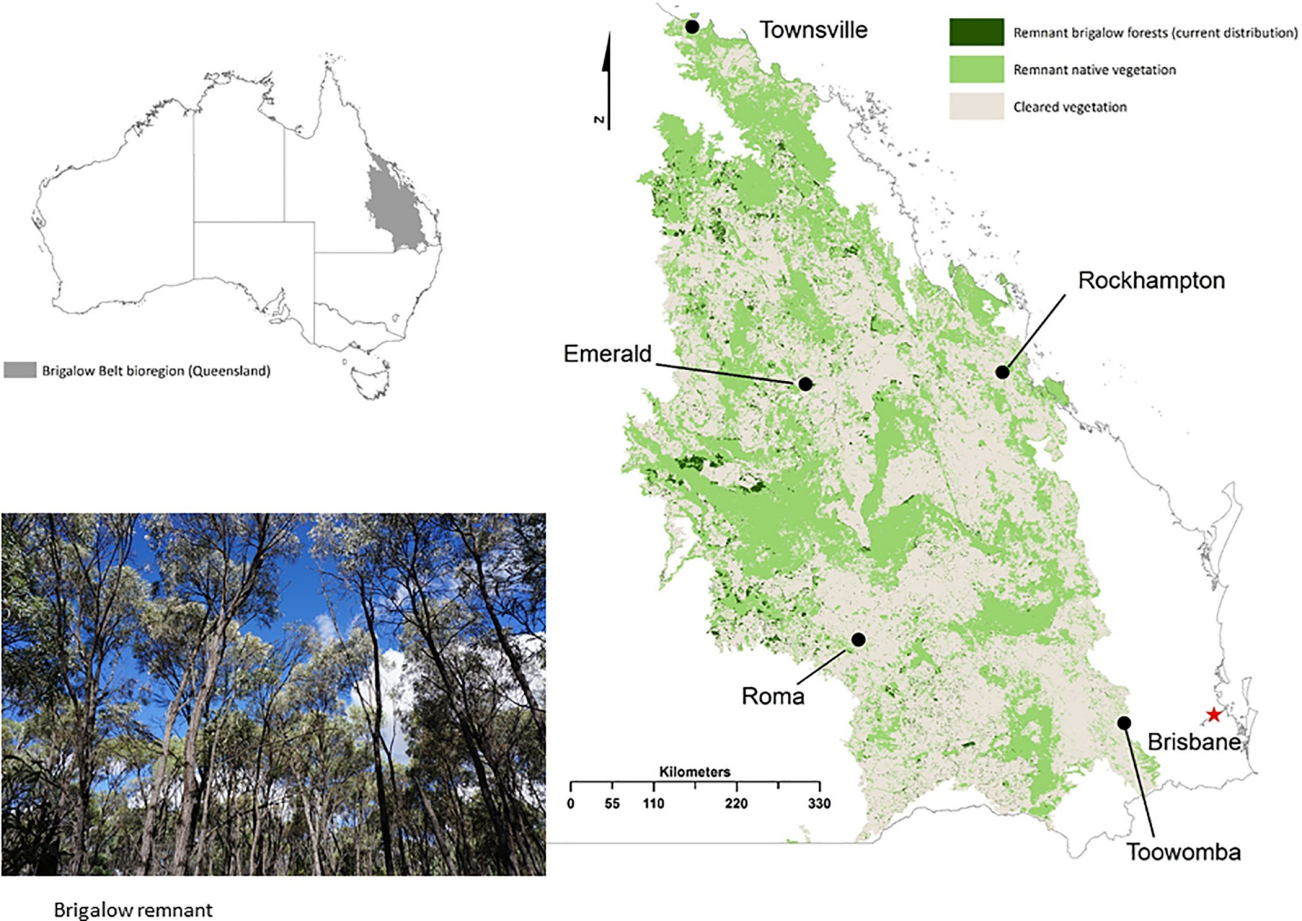
The Brigalow Belt bioregion. Our study region is located in central and north Queensland, Australia. It covers a total area of about 35 million ha, almost as big as Germany. This bioregion is named after the region’s once-dominant tree species, *Acacia harpophylla* F. Muell. ex Benth. It has been estimated that since mid-19^th^ century, 7 million ha of brigalow forest (92% of its original extent) have been cleared in the bioregion. Source: Environment and Science, Queensland Government, Remnant vegetation cover—2015—Queensland, licensed under Creative Commons Attribution 4.0 2015.

The Brigalow Belt () is a large multiple land-use region in central and north Queensland, Australia (- 21.98°S, 148.12°E). It has experienced one of the most rapid landscape transformations ever documented [30], where only a few pristine remnants persist and the majority of the native vegetation is degraded or has been cleared [31] (Fig 2). Since European settlement, a widespread conversion of forests and woodlands to pasture for livestock grazing and cropping [32] has reduced the extent of endemic brigalow forests and woodlands to only 8% (600 000 ha) of its original 7.3 million ha extent. The key threats to the Brigalow’s biodiversity are the cumulative impacts of the diverse regional land-uses: broad scale clearing, fragmentation and modification of native vegetation for agricultural development [33], urbanisation [34]; logging [35], livestock grazing [36] and most recently, large-scale coal, oil and gas development, including coal seam gas [28]. Invasions of exotic flora and fauna, altered fire regimes, changes to groundwater extraction and shifting climatic conditions are also negatively impacting the condition of the bioregion and its species [37]. Despite the many threats affecting the Brigalow Belt, it is formally recognised as a biodiversity hotspot [22], notable for its fauna. It supports the highest bird diversity of any Australian bioregion (328 resident species in the Brigalow Belt South) and a rich herpetological fauna (148 species or about 20% of Australia’s reptiles), including several endemic species [38]. Mammals are the most threatened taxonomic class in this bioregion, with nine of 14 species listed as critically endangered, endangered or vulnerable to extinction (see Supplementary Information). The Brigalow bioregion supports 172 regional ecosystems; 40 of these ecosystems are listed as endangered, and 62 as ‘of concern’ [39].

### Data collection

We collated information to support informed conservation priority setting in the Brigalow Belt from existing databases and literature, supplemented with a structured expert elicitation approach [40, 41]. We sought participation from a broad and representative group of stakeholders and experts in the biodiversity, conservation and management of the Brigalow Belt region, and forty people participated in the study. Ethics approval was obtained from the CSIRO Social and Interdisciplinary Science Human Research Ethics Committee (CSSHREC) before this study began. Participants were from Queensland universities and CSIRO (9), state government employees (15), private landholders (2), non-government conservation organisations (2), regional natural resources managers (4), gas extraction industry (3) and private environmental consultants (5).

During a three-day elicitation workshop and a follow-up consultation process, participants developed and agreed on 11 management strategies plus a common vision to enhance the functional persistence of 77 endangered fauna species and 102 endangered flora species over the next 50 years (179 species in total). For the flora species, participants provided also the ecosystem where the species are found. A functionally persisting species exists at high enough population sizes to achieve their ecological function over the next 50 years [42]. For a detailed description of the selected species, their listing category and the proposed management strategies with their related actions and costs, see Supplementary Information (S1 and S2 Tables). Strategies were proposed by participants in discussion groups of 4–5 people to minimise bias from stronger personalities, ensuring all participants could have their say. The strategies were discussed by all participants and agreed upon using a consensus method, where disagreements were resolved with constructively facilitated discussions. We followed the elicitation approach described in [24].

Participants re-formed into groups of 3–8 people, based on the strategies that they had expertise to parameterise. For each management strategy they were asked to define a specific goal, a set of actions necessary to achieve the goal, estimates of any costs (in Australian dollars) and information required to estimate feasibility of implementation value over the 50 year time frame (Fig 2C and S2 Table). Actions are discrete activities needed to achieve the goals of each strategy, and may involve on-ground management, planning, education, data collection, mapping, research, and communication activities. Participants used their existing knowledge and other accessible information (management reports etc.) to estimate the costs of each action over the 50 year period, using prompts of the kinds of costs that could be incurred. If participants did not have the necessary information to cost out an action, they listed people or reports to follow up with post workshop. Expected costs were converted to net present costs (total expected cost over 50 years in present day terms) and annualised average cost (average expected cost/year in present day terms) using a discount rate of 7%, the recommended rate for public investments in Australia [43]. Using annualised average costs facilitate communication and comparisons of strategies with sequencing of actions over different time spans [28]. Participants were then tasked with two critical questions: first, the feasibility of each action being implemented, and second, the biodiversity benefits that would result from effective implementation of those actions. Participants worked individually and anonymously to minimise the potential for biases that occur when estimation is carried out in groups [44, 45]. This process was followed by group discussions. Finally, participants were given an opportunity to revise their estimates anonymously based on the group discussion.

To estimate the feasibility of each action, participants were asked to use a likelihood scale [41] (S2 Fig) to provide the probability of uptake (the likelihood that the action would be implemented, considering the economic, social and political factors at the time) and the probability of success of the action (the likelihood that if implemented the action would be effective). For example, an action to create a firebreak around a sensitive vegetation pocket would first be subject to a probability of uptake (the proportion of landowners would agree to create the fire break) and a probability of success (if created, how likely is it that the fire break would effectively protect the sensitive vegetation, e.g. could the fire move past the break under windy conditions, etc.). The feasibility of each action was then calculated using the product of the probability of uptake and the probability of success of each action. We averaged the feasibility values across all actions within a strategy to estimate the overall feasibility of each strategy [25]. Participants were then asked individually to estimate the improvement in feasibility (as a percentage) that they believed a common vision would generate. From this we calculated an overall feasibility with the common vision for each strategy.

Benefits were measured as improvements in the probability of functional persistence with and without the implementation of each of the 11 on-ground management strategies. Following a modified Delphi approach that is designed to minimise expert overconfidence [46], workshop participants gave a four-point estimate (estimates of the lowest, highest, best case estimates, and their confidence that the true value would be between the lower and upper bounds) of the probability of functional persistence of each species. The four-point estimate method [46] asks for a confidence interval in the following way:

Given the evidence you have,

- Realistically, what do you think is the lowest persistence probability value of management strategy X on species A could be?
- Realistically, what do you think is the highest persistence probability value of management strategy X on species A could be?
- Realistically, what is your best guess (e.g. most likely estimate)?
- How confident are you that the actual persistence probability value of management strategy X on species A is between your lower and upper estimates? Please enter a number between 0–100%.

Following the workshop, the participants were invited to anonymously revise their estimates considering the responses of other participants. Estimates were made under a baseline scenario (i.e. without considering the implementation of any of the proposed management strategies) and then under each of the 11 management strategies. The benefit of each strategy was then calculated as the difference in the persistence value under that strategy compared with the baseline scenario, as described below.

### Analysing the cost-effectiveness of strategies

Cost-effectiveness prioritisation can be used to assess strategies independently based on their cost-to benefit-ratio, where the benefit is measured in non-monetary terms [47]. We estimated the cost-effectiveness of the strategy *i* (*CE*_*i*_) as the potential benefit of the strategy (*B*_*i*_) multiplied by the feasibility (*F*_*i*_) divided by the expected cost (*C*_*i*_).

$${CE}_{i}=\frac{B_{i}F_{i}}{C_{i}}.$$ (1)

The potential benefit *B*_*i*_ of implementing strategy *i* is defined by the cumulative difference in persistence probability of all threatened species in the region with and without implementation of a particular strategy averaged over the number of participants who made the prediction for the species:

$$B_{i}=\sum_{j=1}^{N}\frac{\sum_{k=1}^{M_{j}}\left(P_{ijk}-P_{0jk}\right)}{M_{j}},$$ (2)

where *P*_*ijk*_ is the probability of persistence of species *j* if strategy *i* is implemented (as estimated by expert *k*); *P*_0*jk*_ is the probability of persistence of species *j* if no strategy is implemented (baseline scenario; as estimated by expert *k*); *N* is the number of threatened species; and *M*_*j*_ is the number of participants who made predictions for species *j*. We estimated the likely improvement in persistence across fauna species, flora species and all species combined in the region if a particular strategy was implemented.

Building the common vision also incurs a cost, using resources that could otherwise be spent on the on-ground management strategies. This raises the question of whether it is worth investing in a common vision. So, before proceeding to develop the vision, it is necessary to evaluate its expected return on investment along with on-ground management strategies. To do this, we ran the cost-effectiveness analyses twice, assuming strategies were implemented with and without a common vision. For the scenarios where a strategy is implemented with the common vision, we added the cost of the common vision to the cost of each strategy and recalculated the cost-effectiveness of each strategy using the improved feasibility values estimated by the participants assuming a common vision was implemented (Fig 2 and S3 Table). If the cost-effectiveness score of an action is higher when using the feasibility and costs that assume the common vision is implemented, then the common vision is a worthwhile investment. We also conducted uncertainty analyses to investigate the contribution of the parameters in the cost-effectiveness model; and assessed the robustness of the cost-effectiveness priority order to the uncertainty of the participants’ predictions (Supplementary Information).

### Estimating the threshold break-even value of the common vision

As there is some uncertainty around the cost of the common vision, i.e. it may end up being costlier than estimated, we determined the threshold break-even value at which it would become worthwhile to invest in a common-vision. The threshold break-even value of the common vision was calculated by equating the cost-effectiveness of the strategies (Eq 1) with and without the common vision. Letting *B* represent the benefit of the strategy (equivalent with and without the common vision), *F*_*CV*_ and $F_{\bar{CV}}$ represent the feasibilities with and without the common vision respectively; *C*_*CV*_ represent the cost that could be spent implementing the common vision, and $C_{\bar{CV}}$ represent the cost of management without the common vision, we have:

$$\frac{BF_{CV}}{C_{CV}+C_{\bar{CV}}}=\frac{BF_{\bar{CV}}}{C_{\bar{CV}}}$$ (3)

Our objective was to find *C*_*CV*_, the cost that could be spent on the common vision while maintaining the same cost-effectiveness ratio as implementing the strategy with no common vision. Re-arranging, we obtain:

$$C_{CV}=\frac{F_{CV}}{F_{\bar{CV}}}C_{\bar{CV}}-C_{\bar{CV}}$$ (4)

*C*_*CV*_ provides an upper limit for cost-effective spending on the common vision. Spending up to *C*_*CV*_ on the common vision would result in greater cost-effectiveness from investing in the proposed strategy than implementing the strategy without the common vision. In contrast, spending more than *C*_*CV*_ on the common vision would result in a lower cost-effectiveness than implementing the strategy alone. For example, implementing a pest control program in collaboration with a wide range of landholders is likely to be costlier (involving additional coordination and so forth) but more effective than a publicly implemented program in isolation.

### Identifying complementarity sets of strategies under different budgets

Cost-effectiveness ranking analysis provides information on the relative return on investment of each strategy but does not provide the best combinations of strategies for investing limited funds under a given budget. To provide this information, we applied an optimisation approach using a linear programming formulation and CPLEX version 13.0 [48] to find the combination of complementary set of strategies that secured the most species per unit cost over a range of set budgets. A ‘secure’ species was defined as a species that is estimated to persist with a probability that exceeds a fixed persistence threshold over 50 years. We investigated three persistence thresholds (50%, 70% and 90%) over a range of budget levels [49].

Our optimal solutions are Pareto optimal solutions [49]. The set of strategies that maximised the number of species above a persistence threshold (*τ*) while minimising the strategies’ implementation cost was found using:

$$\max\;\sum_{i\in S}\sum_{j\in N}p_{ij}x_{i}\;\mathrm{and}\;\min\;\sum_{i}C_{i}x_{i}$$ (5)

**Fig 3:**
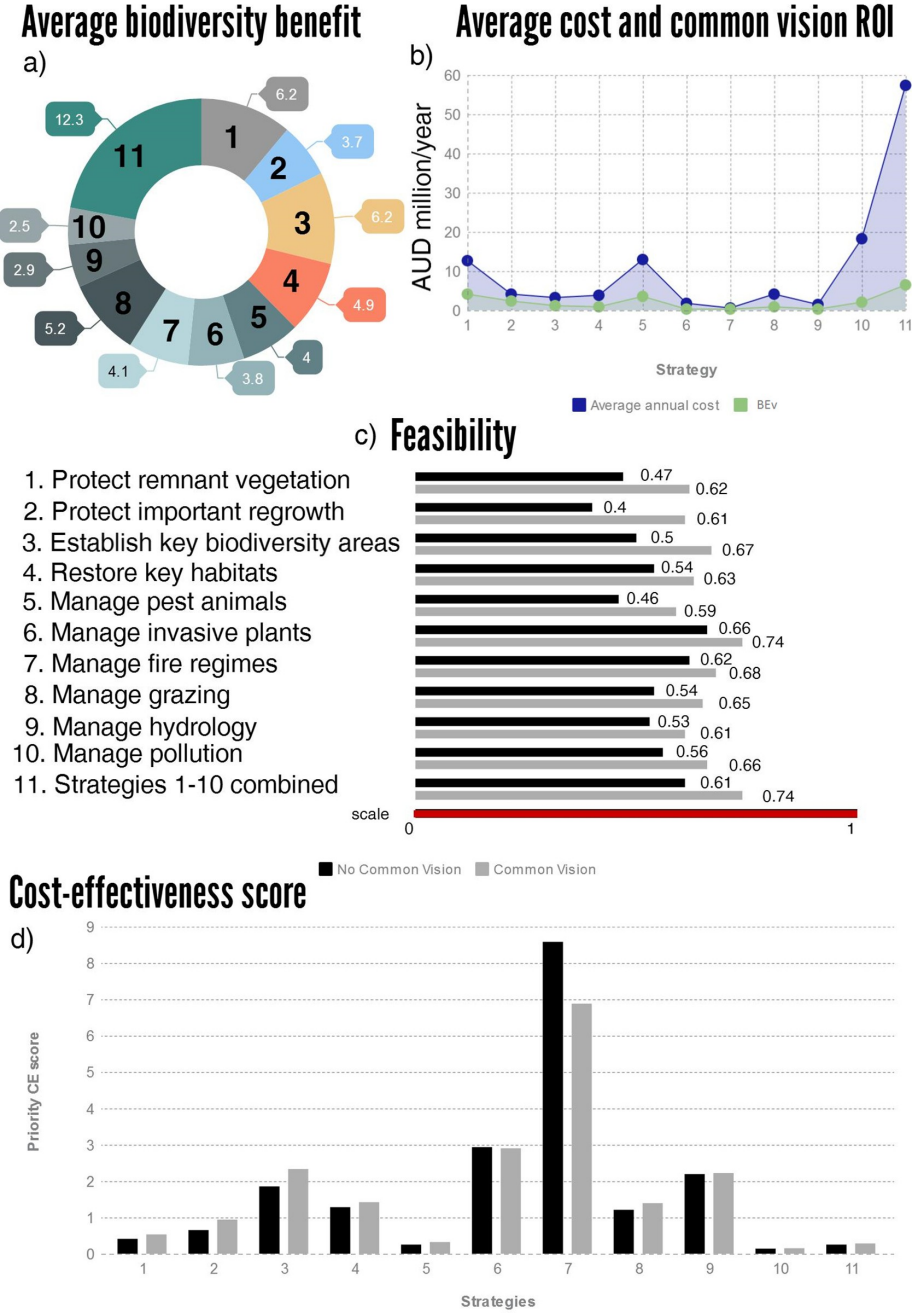
Key conservation strategies appraisal for threatened species across the Brigalow Belt bioregion in Queensland. a) average potential benefits (percentage improvement in species persistence averaged across all species for each management strategy); b) annualised average costs of each strategy and threshold break-even value (BEv) of the common vision when implemented with each strategy; c) feasibility values (0–1) with and without the common vision; d) cost-effectiveness (CE) of each management strategy for all species combined with and without the common vision.

Where *x*_*i*_ denotes whether a strategy (*x*_*i*_ = 1) or not (*x*_*i*_ = 0) is included in the optimal set of strategies. A vector x∈{*x*_1_,*x*_2_,…*x*_*s*_} represents a combination of selected strategies. *S* is the total number of strategies listed in Fig 3; *p*_*ij*_ identifies whether species *j* is expected to reach a given persistence threshold if strategy *i* is implemented; if the expected benefit of applying strategy *i* for species *j* is above the persistence threshold (i.e. *B*_*ij*_*F*_*i*_+*B*_0*j*_>*τ* with $B_{ij}=\frac{\sum_{k=1}^{M_{j}}\left(p_{ijk}-p_{0jk}\right)}{M_{j}}$) then *p*_*ij*_ = 1; on the contrary, if this threshold is not exceeded *p*_*ij*_ = 0. M represents the number of participants who provided persistence estimates for species *j*.

We ran the optimisation analysis twice, with and without accounting for the costs and improved expected benefits resulting from implementing the common vision. We then compared the differences in the number of species and the number of actions that could achieve each of the persistence thresholds with and without the common vision, across the full range of budgets. We also aggregated the species into groups and compared the number of species/ecosystems within each group that met the thresholds for the no common vision and common vision approaches.

## Results

### The common vision strategy and its impacts on conservation management

Building and implementing a regional common vision was proposed as an enabling ‘overarching’ strategy that could be implemented together with any or all of the 11 on-ground strategies (S3 Table). The participants described the core characteristics of this common vision as: to be driven by local stakeholders from all sectors, to form a working group that could help mobilise key experts, with a participatory, bottom-up leadership style; all stakeholders should have a fair say in the vision regardless of their economic contribution to the region; and stakeholders need to compromise to minimize negative impacts across all stakeholders (S2 Table). According to our expert elicitation process, a common vision would improve the likelihood that each of the management strategies is successfully implemented, because of better cohesion, knowledge, co-ordination and participatory decision-making processes. As described by the participants, the common vision for the region was estimated to cost AUD$3M over three years (or an average annualised cost of $0.2m per year over 50 years), spent largely during the first three years and becoming self-sustaining afterwards and should be kept in place over the 50 year duration of management activities considered in our study. Note that we assume the cost of developing the common vision is the same whether it is applied to one or all eleven strategies, in effect assuming that the key costs are in forming the relevant collaboration rather than in reaching consensus for additional strategies.

Investing in collaborations such as building a common vision does not have direct benefits to biodiversity in terms of increasing species persistence. However, participants indicated that it would improve the feasibility of on-ground management strategies, which indirectly results in increased expected benefits. In our Brigalow Belt case study, the range in feasibility of the management strategies increased from 0.40–0.66 without the common vision, to 0.59–0.74 if the common vision was implemented (Fig 2C). This improved the average expected benefit (increase in the species persistence) of these management strategies by 9–52% depending on the strategy (S3 Table).

### The cost-effectiveness of a common vision and on-ground management strategies

Strategies differed in terms of their relative cost-effectiveness in contributing to species conservation outcomes (Fig 3 and S3 Table). We concluded that managing fire and invasive plant species were the two most cost-effective on-ground strategies for improving the persistence of the Brigalow Belt’s threatened flora and fauna species over the next 50 years (Figs 3D and S1). The priority order of strategies was similar when considering the fauna species. Twenty-one of the 179 threatened species in the Brigalow Belt had greater than a 50% risk of being functionally lost from the region over the next 50 years if no threat management strategies were implemented only, flora species only and flora and fauna combined (S3 Table). Uncertainty analysis (S1 Appendix) showed that the priority order of cost-effectiveness of the strategies was reasonably robust to variability in the persistence estimates, both with and without the common vision (S1 Fig). Fire management was consistently the top priority in terms of cost-effectiveness even though participants expressed the highest levels of uncertainty in terms of the benefits of this strategy for increasing the persistence of threatened species over 50 years (S1 Fig).

Our analysis indicated that implementing the common vision with on-ground management strategies increased the cost-effectiveness of almost all of the strategies but made little difference to their cost-effectiveness ranking order (Fig 2C and 2D, S3 Table). The only scenarios for which the implementation of the common vision was not a worthwhile investment, assuming it costs $0.2m/year, were the implementation of the two most cost-effective strategies: strategies 6 (managing invasive plants) and 7 (managing fire regimes). This was likely due to the comparatively low implementation cost of these two strategies ($0.5m/year and $1.5m/year), meaning that the ratio of the costs of these strategies to the cost of the common vision were lower than other strategies, i.e. the common vision had a lower relative impact on the overall cost of the less expensive strategies (S3 Table).

### The maximum cost-effective investment in building a common vision

Given that cost estimates for conservation strategies are uncertain, we used our approach to determine the maximum amount of funds that could be spent on the common vision before it ceased to be cost-effective for that strategy alone, i.e. the threshold break-even value. The threshold break-even value of the common vision ranged from 9–52% in the proportion of total available funds, in line with the proportion of additional benefits generated by the common vision when coupled with each strategy (Fig 2B; S3 Table). In almost all cases, the threshold break-even value of the common vision was substantially more than the $0.2m/year that it was expected to cost to implement, indicating that the common vision is likely to be a cost-effective investment even if it was more expensive than predicted here. For example, managers could justify spending up to $3.9m/year on the common vision if it was implemented along with the strategy to protect remnant vegetation (Fig 2B and S2 and S3 Tables). Similarly, a threshold of 11% of the available budget ($6.4m/year) could be spent on the common vision if all strategies (1–10) were implemented (Fig 2B and S2 and S3 Tables).

### Complementary sets of strategies to secure species

**Fig 4:**
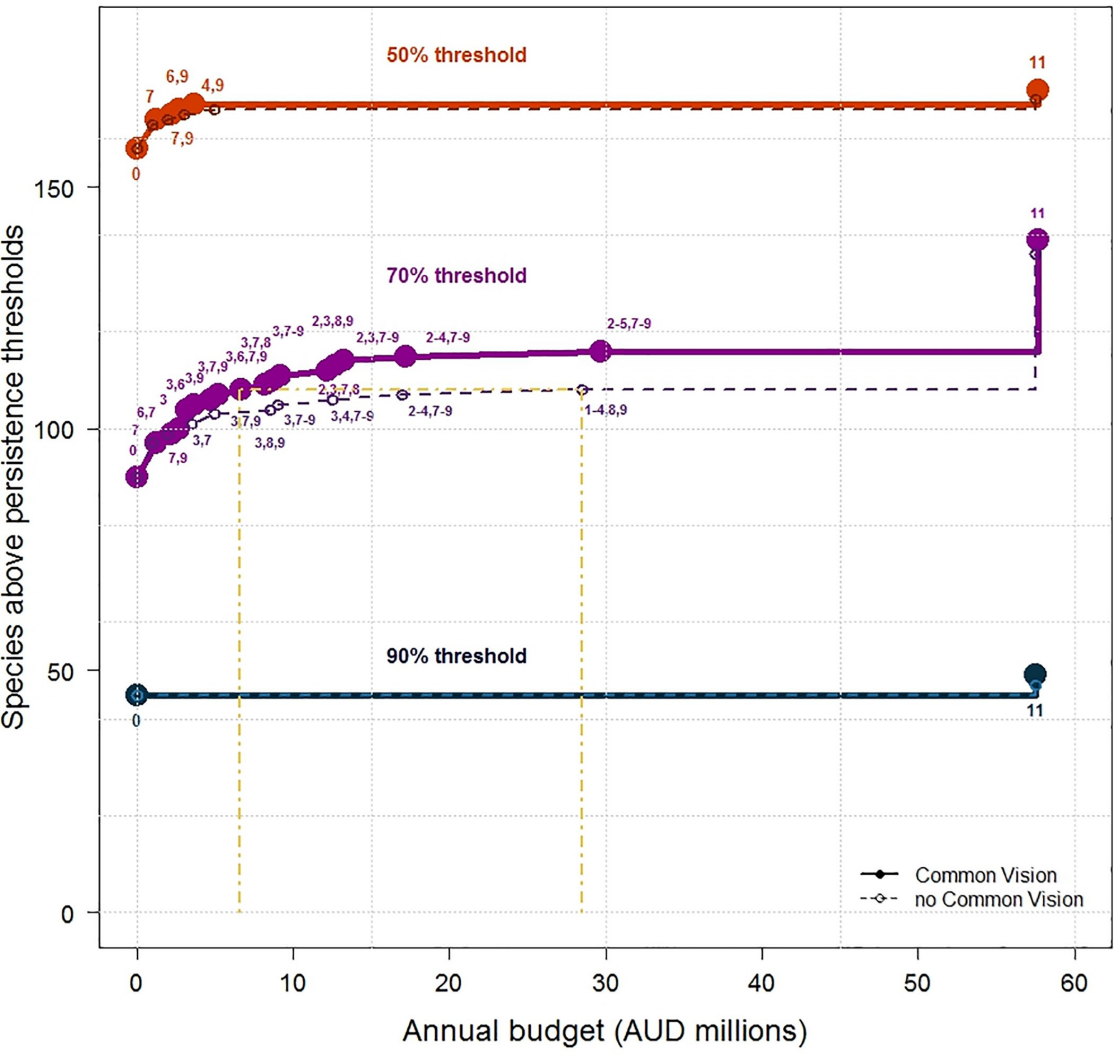
Number of species above different persistent thresholds: 50% (orange), 70% (purple) and 90% (indigo) with the common vision (solid lines) and without the common vision (dashed lines) considering different levels of investment optimally and effectively spent on specific threat management. The numbers above the points represent the combination of strategies (S3 Table). For the 50% and 90% thresholds the same bundles of strategies were selected at each budget with and without the common vision. For the 70% threshold only the first two strategies (7 and 7, 9) coincided with and without the common vision. Yellow dashed lines show the difference in cost of securing 108 species to a 70% probability of persistence without the common vision ($28.5 m/year) compared to only $6.5 m/ year if the common vision is implemented.

Since the highest ranked strategies in a cost-effectiveness analysis may benefit the same set of species, we used a complementarity analysis to select the combinations of strategies that are estimated to ‘secure’ as many species as possible at a range of budgets (Fig 4).

Twenty-one of the 179 threatened species in the Brigalow Belt had greater than a 50% risk of being functionally lost from the region over the next 50 years if no threat management strategies were implemented (Table 1 and S4 Table). Implementing the threat management strategies outlined here, could ‘secure’ (increase the likelihood of persistence to at least 50%) 10 of those species, including the iconic koala (*Phascolarctos cinereus*). The common vision plays a critical role in securing two additional species, Allan’s skink (*Lerista allanae*) and Boggomoss snail (*Adclarkia dawsonensis*) which only reach the 50% threshold if the common vision is implemented (Table 1 and S4 Table). The remaining nine species, including the northern hairy-nosed wombat (*Lasiorhinus krefftii*), the brush-tailed rock-wallaby (*Petrogale penicilliata*), are not expected to be secured with threat management alone, and would require targeted species-specific management to ensure their survival (Tables 1 and S4). If all management strategies including the common vision were implemented, 139 of the 179 (77%) of species could reach or exceed a 70% persistence threshold, indicating a greater level of security (Supplementary Information). Even if all strategies were implemented in the Brigalow Belt region, only 49 of its 179 (26%) threatened species were likely to increase above the 90% persistence threshold (Tables 1 and S4), indicating the challenge of species recovery in highly developed regions.

**Table 1:** Number of species by category or ecological community reaching the each of the persistence thresholds (50, 70 and 90%) with and without the common vision (CV and No CV, respectively). For more details see S4 Table.

Species/ Ecosystems groups (total n)	Persistence thresholds					
	50%		70%		90%	
	No CV	CV	No CV	CV	No CV	CV
Amphibians (3)	3	3	3	3	0	0
Birds (31)	27	27	16	17	2	2
Fish (7)	7	7	4	4	0	0
Invertebrates (4)	3	4	0	0	0	0
Mammals (14)	10	10	4	5	0	0
Reptiles (18)	16	17	11	12	1	1
Brigalow (8)	8	8	8	8	1	1
Ephemeral wetlands and riparian zones (4)	4	4	4	4	0	0
Grasslands (9)	9	9	8	8	1	1
Notophyll Vine Forests (7)	7	7	7	7	6	7
Open forests and woodlands (41)	41	41	38	38	22	22
Open shrublands and heathlands (8)	8	8	8	8	6	6
Permanent wetlands (4)	4	4	4	4	0	0
Serpentine (9)	9	9	9	9	0	0
Semi-evergreen vine thickets (12)	12	12	12	12	8	9
**Total (179)**	**168**	**170**	**136**	**139**	**47**	**49**

Complementary bundles of strategies that include the common vision (at our estimated cost of $0.2m/yr irrespective of the number of strategies in the bundle) were often able to secure the same number of species at a lower cost than bundles that did not include the common vision (bold and dashed lines respectively in Fig 3). For example, a management goal of securing 108 species above a 70% persistence threshold could be achieved by implementing four strategies (3, 6, 7 and 9 in Fig 3) for ~$6.5M/year if the common vision is implemented. This same outcome would cost $28.5M/year and require the implementation of six strategies (1, 2, 3, 4, 8 and 9 in Fig 3) if the common vision is not implemented (Fig 3 dot-dashed lines). This benefit is more apparent for the 70% persistence threshold because there are more combinations of management strategies (with different feasibility values) that can contribute to reach this threshold than for the other two persistence thresholds (50 and 90%).

## Discussion

Our study demonstrates how expert estimates of increased feasibility can be used to rapidly forecast the return on investment of uniting stakeholders with divergent goals through the development of a common vision. Previous studies quantify the improved effectiveness of stakeholder cooperation retrospectively [20], finding that stakeholder cooperation has improved conservation outcomes [17–19]. Our approach is the first to provide a quantitative estimate of whether these improved outcomes are worth the cost expended, which helps to clarify the value for money of building a common vision or a similar action that has indirect benefits to biodiversity.

In the Queensland Brigalow Belt region, a region as big as Germany, the expected benefits of ‘working together’ were considerable, yet the costs were estimated to be low. The great variety of land-uses and values in the bioregion signifies a diverse group of stakeholders. Building and implementing a common vision in the Brigalow Belt region is crucial to improve biodiversity regional conservation outcomes as it would encourage collaborative decision-making between stakeholders and across the variety of land tenures, according to participants involved with this study. Participants estimated that the common vision would have most impact improving the feasibility of strategies that initially had lower feasibility scores, supporting the notion that collaborations are most important for contentious or challenging situations [7, 8, 50].

Quantitatively, a common vision was estimated to cost less than 1% of the total estimated amount for implementing a comprehensive portfolio of on-ground strategies and while increasing the expected benefit of conservation management investment by 9–52% (S3 Table). Two species (*Lerista allanae* and *Adclarkia dawsonensis*) rely on the implementation of the common vision to reach the 50% persistence threshold (Tables 1 and S4). This indicates that participants believe a common vision is critical for avoiding the loss of natural capital.

Integrating an assessment of overarching and on-ground management strategies into the PTM tool, following our approach, has the advantage of estimating the cost-effectiveness of a common vision before the vision is created, just as the cost-effectiveness of many on-ground management strategies will need to be estimated before they are implemented. The PTM used in this way provides an understanding which and how many species are likely to be protected under various scenarios, which strategies are likely to be the most important for implementation, and the amount of resources needed to allocate to the common vision. However, this approach can be applied elsewhere in the world and at different scales, and, while not yet tested to our knowledge, it could be applied using a range of other conservation decision science tools. The critical factor in being able to assess the cost-effectiveness of a high level strategy such as building a common vision, is that the additional costs and the additional benefit across all the on-ground management strategies are able to be estimated, as a result of building the vision [51, 52]. The overall benefit to cost ratio of scenarios with and without the common vision can then be compared.

The strategies for the Brigalow Belt presented in this study are prioritised for the expected improvement they generate for threatened species only. However, these strategies would also benefit biodiversity more broadly, as well as improve employment, sustainability (improvement in pastoral, agricultural and mining industry practices) and ecosystem services [26, 53]. The priorities could change if different kinds of information are included, if the interactions between strategies are considered to estimate benefits, or if the strategies are only partially undertaken. There are uncertainties in the information used in this study, despite using best practice methods to maximise the quality of elicited information by experts [40, 46, 54]. The best practice approach for implementing priority threat management is through a systematic adaptive process that allows estimates of benefits, feasibility, and costs to be updated progressively over time [23, 26]. Then, it would be necessary to check in, learn, and revise the estimates following any major changes in regional conditions or information availability and at the end of the time frame, at minimum [24]. Further, uncertainties in future conditions (e.g. climate change and future threats or developments) may compound existing threats and accelerate species decline [26]. As a precautionary approach and due to the long management period, the strategies proposed here should be implemented along with actions for vigilantly monitoring emerging threats and adapting management approaches early, and an ongoing review of the effectiveness of implemented strategies [55]. For example, a monitoring program with clear objectives and responses targets that are measurable and representative of the system [56, 57]. Additional knowledge on the cost-effectiveness of building a common vision could be gained by undertaking an impact evaluation, if the common vision and on-ground management strategies defined here were to be implemented, which could reveal much-needed information on the relative importance and management effectiveness of strategies and decision making processes in other regions [58].

The building of a common vision for ecological management in multi-used regions will often be a challenging task, requiring the integration of different priorities, beliefs, and values of the diverse set of stakeholders [59]. Stakeholders may have dramatically divergent objectives and therefore different incentives to participate in management that benefits biodiversity [60, 61]. In regions like the Brigalow Belt, the distribution of power between stakeholders can be unequal, making it challenging to reach a shared vision for the region. If it were to occur, the process should use a well-designed stakeholder collaboration plan to minimise risks [9]. For example, through a neutral third party acting as an arbitrator or mediator, the power between stakeholders can be equalised and the potential manipulation of the more powerful stakeholders could be minimised [62]. Social science tools such as conflict management and resolution, consensus building, and negotiation, could help achieve more effective science-driven targets [63]. A collaborative learning framework, instead of demanding absolute consensus on contentious issues, assists stakeholders to work through issues that constrain the progress towards achieving goals for the common good, by encouraging joint learning, open communication, and constructive conflict management between diverse stakeholders [64]. And finally, an adaptive management framework can help to understand and incorporate the different learnings of resolving involved uncertainties, such as those related to the natural systems or the willingness of people to implement the management strategies [65].

The decision to undertake any action, before implementation occurs, including building and implementing a common vision, must be made based on the best available information. Our approach clarifies the value of a common vision in terms of improving the expected outcomes of conservation actions. Applying the approach could help to define and secure necessary funding for a range of social and ecological-based strategies. It allows stakeholders to prioritise, in a systematic, transparent and science-based way, whether and how much resources to invest in social strategies such as a common vision as part of a management strategy portfolio that meets biodiversity goals.

## Supporting information

S1 Appendix Uncertainty analyses. (DOCX)

S1 Fig Uncertainty plots. (DOCX)

S2 Fig Likelihood scale. (DOCX)

S1 Table Summary table of threatened species. (DOCX)

S2 Table Details of management strategies. (DOCX)

S3 Table Key conservation strategies appraisal. (DOCX)

S4 Table Pareto tables (additional excel files). (XLSX)

## References

[1] Venter O, Sanderson EW, Magrach A, Allan JR, Beher J, Jones KR, et al Sixteen years of change in the global terrestrial human footprint and implications for biodiversity conservation. Nature Communications. 2016;7:12558 10.1038/ncomms12558 27552116 PMC4996975

[2] Butchart SHM, Walpole M, Collen B, van Strien A, Scharlemann JPW, Almond REA, et al Global Biodiversity: Indicators of Recent Declines. Science. 2010;328(5982):1164–8. 10.1126/science.1187512 20430971

[3] Newbold T, Hudson LN, Hill SLL, Contu S, Lysenko I, Senior RA, et al Global effects of land use on local terrestrial biodiversity. Nature. 2015;520(7545):45–50. 10.1038/nature14324 25832402

[4] Game ET, Meijaard E, Sheil D, McDonald-Madden E. Conservation in a Wicked Complex World; Challenges and Solutions. Conservation Letters. 2014;7:271–7. 10.1111/conl.12050

[5] McShane TO, Hirsch PD, Trung TC, Songorwa AN, Kinzig A, Monteferri B, et al Hard choices: Making trade-offs between biodiversity conservation and human well-being. Biological Conservation. 2011;144(3):966–72.

[6] Young JC, Waylen KA, Sarkki S, Albon S, Bainbridge I, Balian E, et al Improving the science-policy dialogue to meet the challenges of biodiversity conservation: Having conversations rather than talking at one-another. Biodiversity and Conservation. 2014;23(2):387–404. 10.1007/s10531-013-0607-0

[7] Boiral O, Heras-Saizarbitoria I. Managing Biodiversity Through Stakeholder Involvement: Why, Who, and for What Initiatives? Journal of Business Ethics. 2015:1–19. 10.1007/s10551-015-2668-3

[8] Phillipson J, Lowe P, Proctor A, Ruto E. Stakeholder engagement and knowledge exchange in environmental research. Journal of Environmental Management. 2012;95(1):56–65. 10.1016/j.jenvman.2011.10.005 22115511

[9] Reed MS. Stakeholder participation for environmental management: A literature review. Biological Conservation. 2008;141(10):2417–31. 10.1016/j.biocon.2008.07.014.

[10] Levin IM. Vision Revisited: Telling the Story of the Future. The Journal of Applied Behavioral Science. 2000;36(1):91–107. 10.1177/0021886300361005

[11] Webler T, Renn O. A brief primer on participation: philosophy and practice In: Renn O, Webler T, Wiedemann P, editors. Fairness and competence in citizen participation: evaluating models for environmental discourse. Dordrecht: Kluwer Academic Publishers; 1995.

[12] Renn O. Participatory processes for designing environmental policies. Land Use Policy. 2006;23:34–43.

[13] Svarstad H, Daugstad K, Vistad OI, Guldvik I. New protected areas in Norway: local participation without gender equality. Mt Res Dev. 2006;26:48–54.

[14] Munton R. Deliberative democracy and environmental decision-making In: Berkhout F, Leach M, Scoones I, editors. Negotiating Change: Advances in Environmental Social Science. Cheltenham: Edward Elgar; 2003.

[15] Young J, Marzano M, White RM, McCracken DI, Redpath SM, Carss DN, et al The emergence of biodiversity conflicts from biodiversity impacts: characteristics and management strategies Biodiversity Conservation. 2010;19(14):3973–90.

[16] Lochner P, Weaver A, Gelderblom C, Peart R, Sandwith T, Fowkes S. Aligning the diverse: the development of a biodiversity conservation strategy for the Cape Floristic Region. Biological Conservation. 2003;112(1–2):29–43. 10.1016/S0006-3207(02)00394-4.

[17] Young JC, Jordan A, R. Searle K, Butler A, S. Chapman D, Simmons P, et al Does stakeholder involvement really benefit biodiversity conservation? Biological Conservation. 2013;158:359–70. 10.1016/j.biocon.2012.08.018.

[18] Margerum RD, Whitall D. The challenges and implications of collaborative management on a river basin scale. Journal of Environmental Planning and Management. 2004;47(3):409–29.

[19] Lane M, Robinson C, Taylor B. Contested country: local and regional natural resources. Victoria, Australia: CSIRO Publishing; 2009. 250 p.

[20] Mazor T, Possingham HP, Kark S. Collaboration among countries in marine conservation can achieve substantial efficiencies. Diversity and Distributions. 2013;19(11):1380–93. 10.1111/ddi.12095

[21] Koontz TMT C. W. What Do We Know and Need to Know about the Environmental Outcomes of Collaborative Management? Public Administration Review. 2006;66:111–21. 10.1111/j.1540-6210.2006.00671.x

[22] Commonwealth of Australia. Australia's 15 National Biodiversity Hotspots. Australia's 15 National Biodiversity Hotspots. Canberra: Australian Government Department of the Environment; 2007.

[23] Carwardine J, O'Connor T, Legge S, Mackey B, Possingham HP, Martin TG. Prioritizing threat management for biodiversity conservation. Conservation Letters. 2012;5(3):196–204. 10.1111/j.1755-263X.2012.00228.x WOS:000305282500005.

[24] Carwardine J, Martin TG, Firn J, Reyes RP, Nicol S, Reeson A, et al Priority Threat Management for biodiversity conservation: A handbook. J Appl Ecol. 2019;56(2):481–90. 10.1111/1365-2664.13268

[25] Chadès I, Nicol S, van Leeuwen S, Walters B, Firn J, Reeson A, et al Benefits of integrating complementarity into priority threat management. Conservation Biology. 2015;29:525–36. 10.1111/cobi.12413 25362843

[26] Firn J, Maggini R, Chadès I, Nicol S, Walters B, Reeson A, et al Priority threat management of invasive animals to protect biodiversity under climate change. Global Change Biology. 2015;21(11):3917–30. 10.1111/gcb.13034 26179346

[27] Firn J, Martin TG, Chadès I, Walters B, Hayes J, Nicol S, et al Priority threat management of non-native plants to maintain ecosystem integrity across heterogeneous landscapes. J Appl Ecol. 2015;52(5):1135–44. 10.1111/1365-2664.12500

[28] Ponce Reyes R, Firn J, Nicol S, Chades I, Stratford D, Martin T, et al. Threat management for imperilled species of the Queensland Brigalow Belt Brisbane: CSIRO, 2016.

[29] Martin TG, Kehoe L., Mantyka-Pringle C, I. Chades SW, Bloom R, Davis S, et al Prioritizing recovery funding to maximize conservation of endangered species. Conservation Letters. 2018 (in Review).

[30] McAlpine CA, Sutcliffe T, Taylor K. One hundred and fifty years of landscape change for two sub regions of the Southern Brigalow: Patterns and management implications In: Franks AJ, Playford J, Shapcott A, editors. Landscape Health of Queensland: Brisbane: Royal Society of Queensland; 2002 p. 27–41.

[31] Butler DW. Planning iterative investment for landscape restoration: Choice of biodiversity indicator makes a difference. Biological Conservation. 2009;142(10):2202–16. 10.1016/j.biocon.2009.04.023.

[32] Accad A, Neldner VJ, Wilson BA, Niehus RE. Remnant vegetation in Queensland. Analysis of remnant vegetation 1997–2009, including regional ecosystem information Brisbane: Queensland Department of Science, Information Technology, Innovation and the Arts (http://www.ehp.qld.gov.au/ecosystems/remnantvegetation/index.html), 2012.

[33] Seabrook L, McAlpine C, Fensham R. Cattle, crops and clearing: Regional drivers of landscape change in the Brigalow Belt, Queensland, Australia, 1840–2004. Landscape and Urban Planning. 2006;78(4):373–85. 10.1016/j.landurbplan.2005.11.007.

[34] Catterall CP, Kingston MB, Park K, Sewell S. Deforestation, urbanisation and seasonality: Interacting effects on a regional bird assemblage. Biological Conservation. 1988;84:65–81.

[35] Maron M, Bowen M, Fuller R, Smith JC, Eyre TJ, Mathieson M, et al Spurious thresholds in the relationship between species richness and vegetation cover. Global Ecology and Biogeography. 2011;21:682–592. 10.1111/j.1466-8238.2011.00706.x

[36] Eyre TJ, Maron M, Mathieson MT, Haseler M. Impacts of grazing, selective logging and hyper-aggressors on diurnal bird fauna in intact forest landscapes of the Brigalow Belt, Queensland. Austral Ecology. 2009;34(6):705–16. 10.1111/j.1442-9993.2009.01979.x WOS:000269675600012.

[37] Ferrier S, Harwood T, Williams KJ. Queensland's biodiversity under climate change: Ecological scaling of terrestrial environmental change. CSIRO Climate Adaptation Flagship Working Paper No. 12B, 2012.

[38] Cogger H, Ford H, Johnson C, Holman J, Butler D. Impacts of land clearing on Australian wildlife in Queensland. WWF Australia, 2003.

[39] Herbarium Queensland. Regional Ecosystem Description Database (REDD). Brisbane: 2015.

[40] Martin TG, Burgman MA, Fidler F, Kuhnert PM, Low-Choy S, McBride M, et al Eliciting expert knowledge in conservation science. Conservation Biology. 2012;26:29–38. 10.1111/j.1523-1739.2011.01806.x 22280323

[41] Morgan MG. Use (and abuse) of expert elicitation in support of decision making for public policy. PNAS. 2013;111:7176–84. 10.1073/pnas.1319946111 PMC4034232 24821779

[42] Carwardine J, O’Connor T, Legge S, Mackey B, Possingham HP, Martin TG. Priority threat management to protect Kimberley wildlife. 2011.

[43] Council of Australian Governments. A guide for ministerial councils and national standard bodies. Canberra, Australia: Australian Government, Department of Prime Minister and Cabinet, 2007.

[44] Burgman MA MM, Ashton R, Speirs-Bridge A, Flander L, et al Expert Status and Performance. PLOS ONE. 2011;6(7):e22998 10.1371/journal.pone.0022998 21829574 PMC3146531

[45] McBride MF, Garnett ST, Szabo JK, Burbidge AH, Butchart SHM, Christidis L, et al Structured elicitation of expert judgments for threatened species assessment: a case study on a continental scale using email. Methods in Ecology and Evolution. 2012;3(5):906–20. 10.1111/j.2041-210X.2012.00221.x

[46] Speirs-Bridge A, Fidler F, McBride M, Flander L, Cumming G, Burgman M. Reducing overconfidence in the interval judgments of experts. Risk analysis: an official publication of the Society for Risk Analysis. 2010;30(3):512–23. Epub 2009/12/25. 10.1111/j.1539-6924.2009.01337.x . 20030766

[47] Levin HM, McEwan PJ. Cost-Effectiveness Analysis: Methods and Applications. Thousand Oaks, California: Sage Publications; 2001.

[48] Tulloch AIT, Chadès I, Dujardin Y, Westgate MJ, Lane PW, Lindenmayer D. Dynamic species co-occurrence networks require dynamic biodiversity surrogates. Ecography. 2016:(Early View). 10.1111/ecog.02143

[49] Nemhauser GL, Ullmann Z. Discrete dynamic programming and capital allocation. Management Science. 1996;15:494–505.

[50] Jones-Walters L, Çil A. Biodiversity and stakeholder participation. Journal for Nature Conservation. 2011;19(6):327–9. 10.1016/j.jnc.2011.09.001.

[51] Parkhurst GM, Shogren JF, Bastian C, Kivi P, Donner J, Smith RBW. Agglomeration bonus: an incentive mechanism to reunite fragmented habitat for biodiversity conservation. Ecological Economics. 2002;41(2):305–28. 10.1016/S0921-8009(02)00036-8.

[52] Wondolleck JM, Yaffee SL. Making collaboration work: Lessons from innovation in natural resource managment: Island Press; 2000.

[53] Carwardine J, Hawkins C, Polglase P, Possingham HP, Reeson A, Renwick AR, et al Spatial Priorities for Restoring Biodiverse Carbon Forests. BioScience. 2015 10.1093/biosci/biv008

[54] McBride MF, Fidler F, Burgman MA. Evaluating the accuracy and calibration of expert predictions under uncertainty: predicting the outcomes of ecological research. Diversity and Distributions. 2012;18:782–94.

[55] Tulloch AIT, Chadès I, Possingham HP. Accounting for complementarity to maximize monitoring power for species management. Conservation Biology. 2013;27(5):988–99. 10.1111/cobi.12092 24073812

[56] Legg CJ, Nagy L. Why most conservation monitoring is, but need not be, a waste of time. Journal of Environmental Management. 2006;78(2):194–9. WOS:000235053300010. 10.1016/j.jenvman.2005.04.016 16112339

[57] Lindenmayer DB, Likens GE, Haywood A, Miezis L. Adaptive monitoring in the real world: Proof of concept. Trends in Ecology and Evolution. 2011;26(12):641–6. 10.1016/j.tree.2011.08.002 21880394

[58] Koontz TM, Thomas CW. What Do We Know and Need to Know about the Environmental Outcomes of Collaborative Management? Public Administration Review. 2006;66:111–21. 10.1111/j.1540-6210.2006.00671.x

[59] Redpath SM, Gutierrez RJ, Wood KA, Sidaway R, Young JC. An introduction to conservation conflicts In: Redpath SM, Gutierrez RJ, Wood KA, Young JC, editors. Conflicts in Conservation: Navigating Towards Solutions. UK: Cambridge University Press; 2015.

[60] Gutiérrez RJ, Wood KA, Redpadth SM, Young JC. Conservation conflicts: future research challenges In: Mateo R, Arroyo B, Garcia JT, editors. Current trends in wildlife research. Switzerland: Springer International Publishing; 2016.

[61] Gray B. Collaborating: Finding common ground for multiparty problems. San Francisco, CA: Jossey-Bass; 1998.

[62] Redpadth SM. Conflicts in conservation: navigating towards solutions. University of Aberdeen, UK: Cambridge University Press; 2015.

[63] Maxwell SL, Milner-Gulland EJ, Jones JPG, Knight AT, Bunnefeld N, Nuno A, et al Being smart about SMART environmental targets. Science. 2015;347(6226):1075–6. 10.1126/science.aaa1451 25745152

[64] Daniels SE, Walker GB. Working Through Environmental Conflict: The Collaborative Learning Approach. Westport, CT: Praeger Publishers; 2001.

[65] Milner-Gulland E. J., Rowcliffe JM. Conservation and sustainable use: a handbook of techniques. Oxford: Oxford University Press; 2007.

