# Supplementary material for: Building a stakeholder-led common vision increases the expected cost-effectiveness of biodiversity conservation
Source: PLoS One. 2019 Jun 13;14(6):e0218093. doi: 10.1371/journal.pone.0218093 (PMC6564421; doi:10.1371/journal.pone.0218093)
Supplement: S1 Appendix — (DOCX) [file pone.0218093.s001.docx]

**S1 Appendix**

Uncertainty analyses

Uncertainty analysis was undertaken to assess how the range of values provided by experts would potentially affect the conclusions of the priority threat management evaluation ^1^. During the elicitation process, each expert provided a most likely, a low, a high persistence probability and a confidence level for each species under each management scenario. The values provided by the experts were used in a Monte Carlo simulation of the model consisting of 10 000 iterations with persistence probabilities generated using beta-PERT distributions given the specified distribution parameters^2^. The values within each iteration were seeded with a random number to draw the value from within the same range given the specified bounds provided by each expert. The outcome of this analysis was a probability distribution of cost-effectiveness of persistence probabilities under each of the management scenarios. These results consider the range of outcomes for each management scenario including the minimum bounds (most pessimistic), maximum bounds (most optimistic) and the mean (most likely) outcomes which are derived from the variability and uncertainty in experts values^2^.

The results of the uncertainty analysis indicate that fire regime management provides the highest level of cost-effectiveness followed by management of invasive plants. More significantly, the most pessimistic benefits associated with fire regime management exceeded the likely optimistic outcomes associated with all other scenarios in terms of the overall cost effectiveness. Implementing management scenarios under a common vision near consistently resulted in enhanced outcomes associated with cost-effectiveness, with the main exception being fire regime management. The results of this suggest that unlike the other management scenarios, fire regime management does not need a common vision to be cost effective when considered as a single management solution.

**References**

1 Cariboni, J., Gatelli, D., Liska, R. and Saltelli, A. The role of sensitivity analysis in ecological modelling. *Ecological Modelling* **203**, 167–182 (2007).

2 McBride, M. F., Fidler, F. & Burgman, M. A. Evaluating the accuracy and calibration of expert predictions under uncertainty: predicting the outcomes of ecological research. *Diversity and Distributions* **18**, 782-794 (2012).
