# Supplementary material for: Building a stakeholder-led common vision increases the expected cost-effectiveness of biodiversity conservation
Source: PLoS One. 2019 Jun 13;14(6):e0218093. doi: 10.1371/journal.pone.0218093 (PMC6564421; doi:10.1371/journal.pone.0218093)
Supplement: S1 Fig — (DOCX) [file pone.0218093.s002.docx]

**
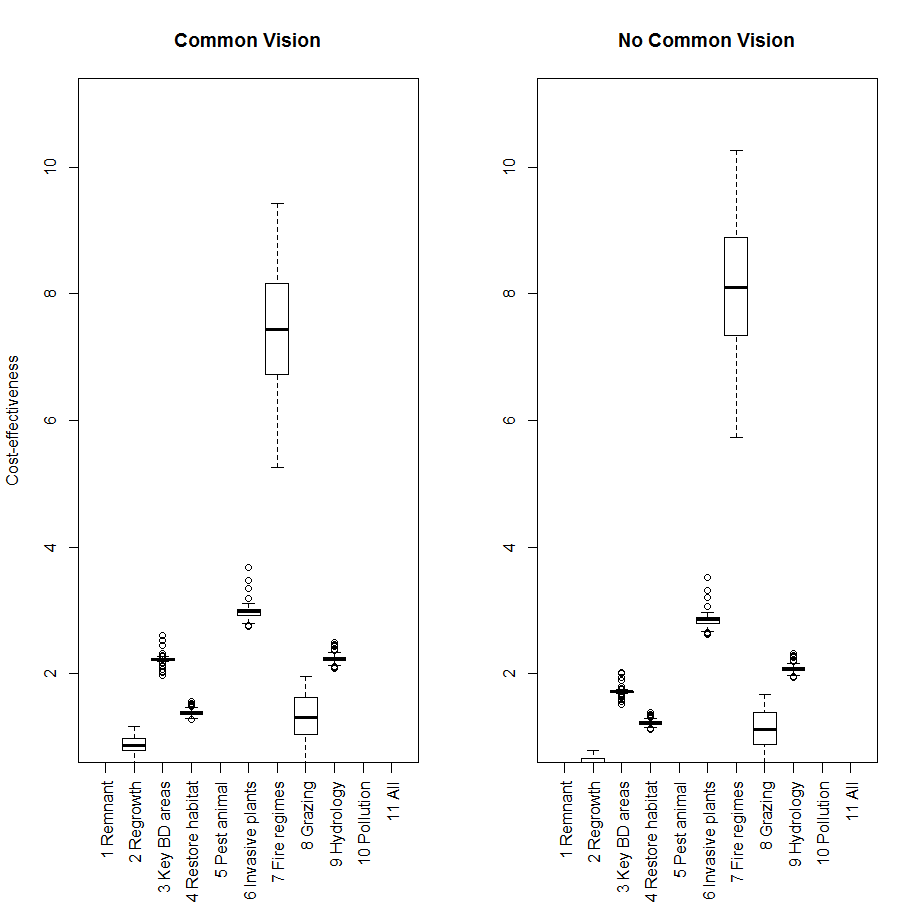
**

**S1 Fig** **Uncertainty plots.** Uncertainty analysis of the cost-effectiveness of each strategy based upon the persistence probabilities for each species as provided during the structured elicitation process.
