# Supplementary material for: Building a stakeholder-led common vision increases the expected cost-effectiveness of biodiversity conservation
Source: PLoS One. 2019 Jun 13;14(6):e0218093. doi: 10.1371/journal.pone.0218093 (PMC6564421; doi:10.1371/journal.pone.0218093)
Supplement: S2 Fig — (DOCX) [file pone.0218093.s003.docx]

**S2 Fig** **Likelihood scales.** The participants were provided with a likelihood scale as a guide to predict the likelihood of strategy being implemented and would be successful if adopted.
