# Supplementary material for: Building a stakeholder-led common vision increases the expected cost-effectiveness of biodiversity conservation
Source: PLoS One. 2019 Jun 13;14(6):e0218093. doi: 10.1371/journal.pone.0218093 (PMC6564421; doi:10.1371/journal.pone.0218093)
Supplement: S1 Table — (DOCX) [file pone.0218093.s004.docx]

**S1 Table** **Summary table of threatened species**. Summary table with the number of species by category that are listed in the Environment Protection and Biodiversity Conservation Act (EPBC), Nature Conservation Act (NCA, Queensland Government), Back on Track (BoT) and Australian Society for Fish Biology - Conservation Status of Australian Fishes (ASFB). CR= Critically endangered; EN= Endangered; VU= Vulnerable; NT= Near threatened; LC=Least concern; H= High, M= Medium and N= None.

|  | EPBC | | | | | NCA | | | | | BoT | | | | ASFB | |
| --- | --- | --- | --- | --- | --- | --- | --- | --- | --- | --- | --- | --- | --- | --- | --- | --- |
|  | CR | EN | VU | LC | N | EN | VU | NT | LC | N | CR | H | M | N | VU | N |
| Amphibians | 0 | 0 | 0 | 0 | 3 | 0 | 1 | 1 | 0 | 1 | 0 | 0 | 0 | 3 |  |  |
| Birds | 1 | 4 | 3 | 0 | 23 | 6 | 7 | 7 | 3 | 8 | 1 | 9 | 1 | 20 |  |  |
| Fish | 1 | 1 | 2 | 0 | 3 | 0 | 0 | 0 | 0 | 7 | 1 | 1 | 0 | 5 | 3 | 4 |
| Invertebrates | 1 | 0 | 0 | 0 | 3 | 1 | 1 | 0 | 0 | 2 | 2 | 1 | 0 | 1 |  |  |
| Mammals | 1 | 4 | 4 | 0 | 5 | 3 | 6 | 1 | 2 | 2 | 4 | 4 | 0 | 6 |  |  |
| Reptiles | 0 | 1 | 6 | 1 | 10 | 4 | 6 | 5 | 2 | 1 | 0 | 7 | 2 | 9 |  |  |
| Brigalow | 0 | 1 | 1 | 0 | 6 | 7 | 1 | 0 | 0 | 0 | 0 | 1 | 0 | 7 |  |  |
| Ephemeral wetlands and riparian zones | 0 | 1 | 1 | 0 | 2 | 1 | 3 | 0 | 0 | 0 | 1 | 0 | 0 | 3 |  |  |
| Grasslands | 0 | 1 | 4 | 0 | 4 | 3 | 6 | 0 | 0 | 0 | 0 | 2 | 0 | 7 |  |  |
| NVF | 0 | 1 | 4 | 0 | 2 | 1 | 6 | 0 | 0 | 0 | 0 | 0 | 0 | 7 |  |  |
| Open forests and woodlands | 0 | 5 | 16 | 0 | 20 | 10 | 30 | 0 | 0 | 1 | 3 | 6 | 0 | 32 |  |  |
| Open shrublands and heathlands | 0 | 1 | 2 | 0 | 5 | 4 | 4 | 0 | 0 | 0 | 0 | 1 | 0 | 7 |  |  |
| Permanent wetlands | 0 | 2 | 0 | 0 | 2 | 3 | 0 | 0 | 0 | 1 | 0 | 2 | 0 | 2 |  |  |
| Serpentine | 0 | 1 | 5 | 0 | 3 | 4 | 5 | 0 | 0 | 0 | 2 | 1 | 0 | 6 |  |  |
| SEVT | 0 | 2 | 6 | 0 | 4 | 5 | 7 | 0 | 0 | 0 | 1 | 3 | 0 | 8 |  |  |
| TOTAL | 4 | 25 | 54 | 1 | 95 | 52 | 83 | 14 | 7 | 23 | 15 | 38 | 3 | 123 | 3 | 4 |
