# Supplementary material for: Building a stakeholder-led common vision increases the expected cost-effectiveness of biodiversity conservation
Source: PLoS One. 2019 Jun 13;14(6):e0218093. doi: 10.1371/journal.pone.0218093 (PMC6564421; doi:10.1371/journal.pone.0218093)
Supplement: S2 Table — (DOCX) [file pone.0218093.s005.docx]

**S2 Table** **Details of management strategies**. Strategies 1-11 are on-ground management strategies. Each strategy has a goal and a set of actions. The cost of each action is expressed as the Annual Equivalent Value (AEV) in Australian dollars (AUD).

| Strategy | Action | Cost (AEV) in AUD |
| --- | --- | --- |
| 1. Protect remnant vegetation | Establish an independent scientific advisory committee to pursue ongoing sustainable management of the region that includes gathering and sharing information, public awareness campaigns and lobbying for legislative changes. | $303,621 |
| Goal: Stop the clearing of native remnant vegetation where possible | Additional funds for driving legislation changes to: stop clearing native vegetation (all remnant and strategic regrowth locations); achieve at least 30% of original extent for each Broad Vegetation Group; connect landscapes; protect key habitats; ensure properties are in good functional condition (30% vegetation on each property, regional vegetation management plan). | $108,557 |
|  | Glossy pamphlet for promotion of best practice land management for biodiversity, using examples of existing success stories. | $175,000 |
|  | Incentive scheme for landholders to retain and manage vegetation. | $12,000,000 |
| 2. Protect important regrowth | Independent committee – as above. | $33,736 |
|  | Additional funds for driving legislation changes – as above. | $0 |
| Goal: Strategically protect regrowth in locations important for biodiversity | Glossy pamphlet – as above. | $0 |
|  | Identify important regrowth locations for protection.  Incentive scheme – as above but at approximately 10% of the cost since regrowth represents 10% of extant vegetation. | $4,000,000 |
| 3. Key biodiversity areas | Survey key sites/ areas to better understand key areas for biodiversity and what they contain. | $1,051,698 |
|  | Incentive scheme for engaging landholders, educating about important areas and securing key biodiversity areas in long-term programs. | $2,225,000 |
| Goal: Identify key biodiversity areas to protect | Monitoring at the landscape level. | $450,000 |
| 4. Restore key habitat | Identify and learn from successful and unsuccessful examples of habitat restored, including key drivers for successes and failures. | $187,500 |
|  | Identify the causes of current state of species and communities-this has been undertaken (SPRAT). | $0 |
| Goal: Restore habitat for key species and communities | Identify and map the location of good candidates for restoration, with consideration of important regrowth locations that require restoration and protection. | $350,000 |
|  | Implement restoration based on situation analysis. | $10,000 |
|  | Allow regrowth / prevent further clearing of Brigalow, SEVT and other vegetation types respecting pre-cleared veg type. | $3,158,000 |
|  | Implement restoration aspects of existing recovery plans. | $1,290,225 |
| 5. Manage pest animals | Map areas of distribution of feral herbivores (rabbits and hares)- this has been undertaken and is available at [www.feralscan.org](http://www.feralscan.org) | $0 |
|  | Bait and rip to 50% or 90% of areas impacted by rabbits and hares (depending upon location). | $76,459.00  $45,875 |
| Goal: Reduce the impact of priority pest animals (rabbits, hares, cats, foxes, wild dogs, ungulates*) on threatened species | Shoot, trap and fence to eliminate (cats and foxes) from strategic locations. | $4,462,756.53 |
| *Experts did not consider cane toads to be a key threat in the region | Shoot, trap, fence and to eliminate feral ungulates from strategic locations. | $1,024,410.00 |
|  | Remove colonies (by shooting) of noisy miners from strategic location. | $3,589,270 |
|  | Undertake complementary restoration action where appropriate (fire exclusion, suckers regeneration, replanting, reducing grazing pressure). | 10,000,000 |
|  | Educate of the value of the dingoes (stop baiting dingoes). | $150,000.00 |
|  | Bait strategically for wild dogs. | $1,500,000.00 |
|  | Avoid internal fragmentation by linear infrastructure inside vegetation and further timber removal (thinning). | $1,000,000.00 |
|  | Create strategic long unburnt habitat landscapes as refugia for small fauna as protection from cats and foxes. | $1,150,000.00 |
|  | Undertake research trials and user experiments to find innovative techniques (e.g. Judas animals/hormonal treatments, corrals, etc). | $2,712,982.59 |
| 6. Manage invasive plants | Understand and manage drivers of weed invasion. Identify and learn from case studies of success/failure and develop a map for areas requiring priority actions. Use soil tests to determine soil condition (landscape management). | $215,849 |
| Goal: Reduce the impact of priority invasive plants on threatened species | Reward system for successful methods for eradicating weeds and showcase good practice by landholders to drive innovation. | $1,037,500 |
|  | Capacity building: improve/train/up-skill existing weed officers. | $185,000 |
|  | Improve wash down station signage on highways. | $1,000,000 |
|  | Develop a Community Of Practice (COP) for threats for everyone to follow (industry especially) to share tips and best practices, and to provide support for each other. Should work with local government authorities. Possibly include COP for nurseries to stop spread of garden-based pests. | $150,000 |
| 7. Manage fire regimes | Develop and implement a coordinated fire management plan for Brigalow Belt bioregion. Manage fire using current knowledge with the interim goal of managing fire frequency, intensity and extent for maximum habitat variety (pyrodiversity) for a suite of fire regimes, i.e. create mosaic of different “age since burnt” habitats. | $600,000 |
| Goal: Manage fire regimes for threatened species | Implement fire management for protection of grassland (including protection of *Tympanocryptis condaminensis*). | $200,000 |
|  | Protect fire sensitive areas. Identify their current state and develop a plan accordingly. | $300,000 |
| 8. Manage grazing | Promote good management (including the economic benefit) through communication. Identify and celebrate “champions”. | $187,500 |
| Goal: Manage grazing and browsing for threatened species | Improve existing best grazing land management practices to include biodiversity. | $150,000 |
|  | Decide on a viable grazing regime to maintain stock routes, road corridors and camping water reserves and communicate to councils. Plans should incorporate how often and how much grazing can occur. | $150,000 |
|  | Identify areas where intensification of grazing should not occur/ be reduced as important for biodiversity and provide incentives for landowners to reduce grazing in these areas. | $5,150,000 |
| 9. Manage hydrology | Develop catchment management strategy that accounts for cumulative impact on biodiversity that includes: a)establish ecological (not only chemical) outcomes and targets to achieve, b)providing statutory agreement to meet ecological targets, c) stop stream diversion, d) determine an control discharge frequency and quality on ephemeral streams and water bodies to replicate natural system. | $825,000 |
| Goal: Manage hydrology for threatened species | Establish long term research program that investigates at the impact on species of concern and ecosystems. Include ongoing periodic monitoring; and research into habitat degradation and cascading effects. | $1,000,000 |
|  | Lobby regulators to avoid mining underneath waterways -especially while reviewing Water Act. | $100,000 |
| 10. Manage pollution | Reduce pollution from agriculture and industry on water that impacts threatened species. | $21,123,708 |
|  | Monitor and feedback. | $5,880 |
| Goal: Manage pollution for threatened species | Develop best management guidelines and extension. | $2,128,000 |
| 11. Combined strategy | Strategies 1-10 combined. | $57,257,087.42 |
| 12. Common vision | Local stakeholders across all sectors to define a shared vision that incorporates environmental, social and economic aspects in a balanced way. | $190,000 |
|  | Establish a “champion” and a core set of people to initiate the vision, but keep it a grass-roots process. |  |
|  | Identify the key people in the best position to drive the shared vision. |  |
|  | Develop and synthesise relevant background information. |  |
|  | Working with a coordinator(s), scope and refine the vision, with representation from all stakeholders. |  |
|  | Communication of vision among sectors. |  |
