# Supplementary material for: Building a stakeholder-led common vision increases the expected cost-effectiveness of biodiversity conservation
Source: PLoS One. 2019 Jun 13;14(6):e0218093. doi: 10.1371/journal.pone.0218093 (PMC6564421; doi:10.1371/journal.pone.0218093)
Supplement: S3 Table — (DOCX) [file pone.0218093.s006.docx]

**S3 Table Key conservation strategies appraisal.** Appraisal of key threat management strategies appraisal for threatened species across the Brigalow Belt bioregion in Queensland: average potential benefits per species for flora, fauna and all combined; feasibility with and without the common vision; net present cost and annualised average costs; cost-effectiveness (CE) priority order and scores for flora, fauna and all species combined; the % increase in expected benefit of the common vision when implemented with each strategy independently, and the maximum justifiable cost of the common vision when implemented with each strategy independently.

| Strategy | Average benefit/species  (%) | | | Feasibility  (0-1) | | Net present cost | Annualised average cost (AAC) | Priority  CE score | | | | Common vision (CV) | |
| --- | --- | --- | --- | --- | --- | --- | --- | --- | --- | --- | --- | --- | --- |
|  |  | | | **No**  **common**  **vision** | **With common**  **Vision** | **$/ 50 years** | **$/year** | **No common vision** | | | **With common vision** | **Increase in expected benefit with CV** | **Maximum justifiable cost of CV**  **$/year (% AAC)** |
|  | **Fauna** n=77 | **Flora** n=102 | **Combined** n=179 |  |  |  |  | **Fauna** | **Flora** | **Combined** | **Combined** | **(%)** |  |
| **1.** Protect remnant vegetation | 8.5 | 4.3 | 6.2 | 0.47 | 0.62 | $171m | $12.4m | 8 (0.25) | 7 (0.16) | 8 (0.41) | 8 (0.53) | 31% | $3.92m (31) |
| **2.** Protect important regrowth | 6.8 | 1.3 | 3.7 | 0.40 | 0.61 | $56m | $4.0m | 7 (0.52) | 8 (0.13) | 7 (0.65) | 7 (0.94) | 52% | $2.12m (52) |
| **3**. Establish key biodiversity areas | 9.0 | 4.0 | 6.2 | 0.50 | 0.67 | $41m | $3.0m | 4 (1.17) | 3 (0.68) | 4 (1.85) | 3 (2.33) | 34% | $1.01m (34) |
| **4.** Restore key habitats | 8.9 | 1.8 | 4.9 | 0.54 | 0.63 | $52m | $3.7m | 5 (1.01) | 6 (0.27) | 5 (1.28) | 5 (1.42) | 16% | $0.59m (16) |
| **5.** Manage pest animals | 7.0 | 1.6 | 4.0 | 0.46 | 0.59 | $178m | $12.7m | 9 (0.20) | 9 (0.06) | 9 (0.25) | 9 (0.32) | 27% | $3.50m (27) |
| **6.** Manage invasive plants | 5.2 | 2.6 | 3.8 | 0.66 | 0.74 | $21m | $1.5m | 2 (1.75) | 2 (1.18) | 2 (2.93) | 2 (2.90) | 11% | $0.17m (11) |
| **7.** Manage fire regimes | 6.2 | 2.4 | 4.1 | 0.62 | 0.68 | $8m | $0.5m | **1 (5.69)** | **1 (2.88)** | **1 (8.58)** | **1 (6.88)** | 9% | $0.05m (9) |
| **8.** Manage grazing | 7.5 | 3.5 | 5.2 | 0.54 | 0.65 | $56m | $4.1m | 6 (0.76) | 5 (0.45) | 6 (1.21) | 6 (1.39) | 20% | $0.82m (20) |
| **9.** Manage hydrology | 4.7 | 1.6 | 2.9 | 0.53 | 0.61 | $17m | $1.2m | 3 (1.56) | 4 (0.63) | 3 (2.19) | 4 (2.22) | 17% | $0.20m (17) |
| **10.** Manage pollution | 4.6 | 0.9 | 2.5 | 0.56 | 0.61 | $252m | $18.2m | 10 (0.11) | 10 (0.03) | 10 (0.14) | 10 (0.15) | 10% | $1.79m (10) |
| **11.** Strategies 1-10 combined | 18.8 | 7.2 | 12.3 | 0.66 | 0.74 | $791m | $57.3m | (0.17) | (0.09) | (0.25) | (0.26) | 11% | $6.44m (11) |
| **12.** Build Common vision |  |  |  |  |  | $3m | $0.2m |  |  |  |  |  |  |
